# Enhancing human resilience beyond COVID-19-related stress: public responses to multi-benefits of home gardening

**DOI:** 10.1038/s41598-023-37426-0

**Published:** 2023-06-29

**Authors:** Chen-Fa Wu, Luu Van Thong Trac, Szu-Hung Chen, Alisara Menakanit, Quoc Tuan Le, Hung-Ming Tu, Chih-Peng Tsou, Hsi-Chih Huang, Nittaya Chookoh, Chih-Cheng Weng, Li-Wei Chou, Chiu-Chuan Chen

**Affiliations:** 1 Department of Horticulture, National Chung Hsing University, Taichung City, 402 Taiwan; 2 Innovation and Development Center of Sustainable Agriculture, National Chung Hsing University, Taichung City, 402 Taiwan; 3 International Master Program of Agriculture, National Chung Hsing University, Taichung City, 402 Taiwan; 4 Department of Horticulture, Kasetsart University, Bangkok, 10900 Thailand; 5 Department of Environmental Sciences, Faculty of Environment and Natural Resources, Nong Lam University - Ho Chi Minh City, Ho Chi Minh City, 700000 Vietnam; 6 Miaoli Management Office, Irrigation Agency, Council of Agriculture, Executive Yuan, Miaoli County, 360 Taiwan; 7 Department of Physical Medicine and Rehabilitation, China Medical University Hospital, Taichung, 404332 Taiwan; 8 Department of Physical Therapy and Graduate Institute of Rehabilitation Science, China Medical University, 406040 Taichung, Taiwan; 9 Department of Physical Medicine and Rehabilitation, Asia University Hospital, Asia University, Taichung, 413505 Taiwan; 10 Department of Landscape Architecture, Chung Chou University of Science and Technology, Changhua County, 510 Taiwan

**Keywords:** Human behaviour, Infectious diseases, Environmental impact, Psychology and behaviour, Lifestyle modification, Population screening, Quality of life, Biological therapy

## Abstract

The SARS-CoV-2 virus has caused a public health crisis globally. Against the backdrop of global resilience, studies have demonstrated the therapeutic value of home gardening as a measure to strengthen human health. However, there is a lack of comparative studies on its benefits across countries. Studies need to examine the role of home gardening in improving public health in various societies to understand and encourage this practice broadly and effectively. We chose Taiwan, Thailand, and Vietnam as case studies, which have suffered substantial pandemic impacts, with millions of infections and thousands of deaths. We explored and compared the perceptions of people on home gardening and its health benefits during the COVID-19 pandemic. We conducted online surveys in three countries between May 1 and September 30, 2022, with a total of 1172 participants. Data were collated on perceived pandemic stress, challenges and solutions in gardening, home gardening intentions, and mental and physical health benefits. In these countries, we found that perceived pandemic stress positively affects home gardening intentions, whereby the motivation of Vietnamese people is the highest. Challenges hinder gardening intentions, while the solutions only positively affect gardening intentions in Taiwan and Vietnam. Home gardening intentions positively affect mental and physical health, whereby there are higher mental health benefits in Taiwanese people than in Thai people. Our findings potentially support public health recovery and promote healthy lifestyles during the COVID-19 pandemic.

## Introduction

Since early 2020, healthcare systems and society worldwide have been severely challenged by coronavirus disease (COVID-19) caused by the SARS-CoV-2 virus, which was declared a Public Health Emergency of International Concern by the World Health Organization in March 2020. COVID-19 outbreaks have infected over half a billion people, posing public health emergency characterized by a concerning escalation in hospitalizations and deaths across countries^1^. Countries of the Asia–Pacific region have been grappling with the impact of COVID-19 outbreaks and enduring severe consequences. Taiwan, Thailand, and Vietnam were once predicted to become the worst-affected countries because of their geographic proximity to China^2–4^. Thailand reported its first case on January 13^5^, followed by Taiwan on January 21^6^, and Vietnam on January 23^7^. These were among the earliest cases of COVID-19 outside of China, where the virus was first identified in late 2019. In recent months, these countries have encountered difficulties due to the emergence of new variants of the SARS-CoV-2 virus, such as the Delta and Omicron variants^8,9^. Through September 2022, Taiwan, Thailand, and Vietnam had reported 6,461,400, 4,681,309 and 11,477,886 confirmed COVID-19 cases, respectively, whereas the total mortality was 11,053 in Taiwan, 32,764 in Thailand, and 43,148 in Vietnam^1,10^.

Despite the challenges, there have been success stories in curtailing the transmission of COVID-19 emerged in Taiwan, Thailand, and Vietnam. In early 2020, one of the key COVID-19 control approaches in Thailand is the “Thai Chana” campaign^11^. At the same time, Taiwanese government implemented multiple COVID-19 control measures, including the mask policy^12^ and “5 K message” is a successful COVID-19 control campaign launched by the government in Vietnam^13^. Governments enforced concurrently measures included stringent lockdowns and social distancing, along with widespread testing and contact tracing, while border controls (e.g., mandatory quarantine) were implemented for incoming international travelers to curb the spread of COVID-19^2,11–13^. Although effective measures were put in place to control the infections, social restrictions also yielded adverse repercussions on the mental well-being of the populace^14,15^. In Taiwan, the social isolation measures significantly constrained the opportunities available to hospitalized patients for familial interaction. These restrictions engendered adverse psychological consequences for patients and their families, such as insomnia and anxiety^14^. Implementing lockdown measures amplified people’s symptoms of insomnia and depression in Thailand, particularly the risk of developing suicidal ideation among individuals displaying depressive symptoms^14,15^. The COVID-19 controls in Vietnam, notably the partial lockdowns and nationwide social distancing, engendered the financial hardships arising from economic disruption during the pandemic^16^. Furthermore, reducing social interactions yielded feelings of isolation, anxiety, depression, and stress, adversely impacting the mental well-being among the populace of Vietnam^16,17^.

COVID-19 pandemic has significantly impacted on human health, both in terms of direct illness and indirect effects. Recent studies reported long-term effects including muscle weakness^18^, reduced physical capacity^19^, and chronic fatigue^20^ in survivors of COVID-19, while physical inactivity during long periods of lockdown or self-isolation also causes comorbid physical health problems (e.g., decreased brain hemodynamics)^21,22^. Physical health should be a long-term concern associated with mental health disorders^22,23^. The typical psychological impacts of COVID-19, including persistent stress disorder^24^ or anxiety and depression^22,25^, have been reported in the mass population. Depressive symptoms may manifest in individuals experiencing post-COVID-19 syndrome^26^, potentially influenced by the systemic inflammation elicited by COVID-19 that exert detrimental effects on the central nervous system^27^ and contribute to the development of depression^26,28^. Around the world, people are grappling with job uncertainty^29^, the rapid spread of fears of infections^16,30^ or worry about vaccine shortages^31^, and lack of effective treatments^32^, also contributing to depression. Studies have shown a significant increase in mood distress, depression, and anxiety disorders arising from unemployment^33^ and isolation^34^ during the pandemic. COVID-19-related lockdowns adversely impacted brain hemodynamics associated with increased mental health disorders (e.g., decreased cognitive functions or depression)^22^. Unemployment causes financial instability and a sense of aimlessness^29^, whereas self-isolation engenders loneliness due to reduced social interactions^35^. Psychological distress can precipitate or exacerbate somatic manifestations affecting physical well-being (e.g., tension headaches, eating disorders, and body aches)^36^ and physical symptoms resembling COVID-19 infection can lead to detrimental consequences on mental health status^37^. Enhancing both the psychological and physical dimensions of public health has become as a research priority during the COVID-19 pandemic^37,38^. It is necessary to be cognizant of the importance of daily routines and physical activities to maintain physical and mental well-being across populations^39,40^.

Exposure to green space has long been recognized as a method to capitalize on nature to boost human health benefits^22,41,42^. Studies have revealed the importance of green spaces to outdoor activities such as recreation^43^ and therapy measures^42,44^. Evidence suggests that human health outcomes can be noticeably improved associated with the green-related affective benefits in terms of mental and physical aspects^45,46^. Urban green spaces have the potential to mitigate unfavorable neuropsychological impacts of COVID-19-related lockdowns and benefit brain health by fostering mental well-being and preventing cognitive decline^22,42^. However, strict epidemic prevention measures, especially the quarantine and social distancing implemented in Taiwan, Thailand, and Vietnam, often resulted in unpleasant experiences^14,16,17^, notably restricting the usage of public green spaces such as parks and urban forests to limit close contact among individuals^47–49^. Although the pandemic prevention policies have effectively limited the spread of infectious cases, closing to nature is an existing demand to maintain human well-being^45^.

Home gardens have potentially become more important than ever in COVID-19 recovery plans^50–52^. The term “home gardening” pertains to the cultivation of various types of plants, such as fruits, vegetables, and herbs, in a garden located within a person’s residential property. This activity can be conducted through diverse settings, including using small containers placed on a balcony, constructing raised beds in a backyard, or joining a community garden^53,54^. Home gardening has been increasingly recognized as an ideal indoor activity allowing people to be close to nature and simultaneously comply with social distancing measures. Home gardening provides multiple benefits, such as reducing stress, depression, and anxiety and improving physical fitness^50^. For example, people convert their home space into a vegetable garden and can be self-sufficient in food against the disruption in the food supply chain^52^. Maintaining food availability benefits households psychologically and ensures nutritional security during the COVID-19 pandemic^52,55^. Additionally, several studies have reported significant physical health benefits such as better sleep quality and improved muscle strength when people have opportunities for physical activity in natural environments^50^.

Many studies have made great efforts to understand the relationship between perceived stress and home gardening that eliminates adverse pandemic effects and improves mental and physical health during the COVID-19 pandemic^45,50,56^. While growing evidence shows physical and mental health benefits from home gardening, there is a lack of comparative studies that examine the impacts of home gardening on human health in different social contexts. Additionally, studies need to examine the specific mechanisms by which home gardening contributes to improved health outcomes and investigate the potential barriers and different solutions to engaging in home gardening. Bridging these gaps in the literature could have significant implications for developing policies and programs aimed at promoting home gardening to enhance public health in diverse global contexts during the COVID-19 pandemic.

Our study seeks to understand the perceptions of people on home gardening and its associated health benefits during the COVID-19 pandemic, focusing on Taiwan, Thailand, and Vietnam. In recent years, home gardening has received more interest in these countries^56–58^. Various homegrown plants and gardening techniques were employed according to their respective geographical distances and climate conditions (e.g., the tropical and subtropical climate)^59,60^, diversifying home gardening practices across countries. However, the choice of home gardening as a health-enhancing activity to reduce stress has just recently gained attention in Taiwan in the context of the COVID-19 pandemic^56^, while there is a lack of evidence supporting its advantages during prolonged pandemic outbreaks in Thailand and Vietnam. Due to the heavy impacts of COVID-19^1,10^, we recognized the necessity to investigate the potential benefits of home gardening to improve people’s physical and mental well-being in these countries during the pandemic. Moreover, implementing distinct strategies and policies to manage the COVID-19 pandemic has yielded divergent outcomes in each country^14,15^, simultaneously they experienced different consequences, including variations in inflections and deaths during the COVID-19 pandemic^1,10^. Therefore, we aimed to examine whether home gardening and its associated health benefits would vary across countries characterized by different restriction levels and degrees of COVID-19 impact. In general, our objective was to clarify and compare the relationship between people’s perceived stress and gardening intentions between Taiwan, Thailand, and Vietnam. We also appraised how people’s exposure to green spaces improves their physical and mental health against the effects of the COVID-19 pandemic. Examining the role of home gardening in promoting health in these countries could provide insights into the potential benefits of this practice for public health more broadly, particularly in the context of COVID-19 pandemic.

We developed five hypotheses to explain the relationship between perceived stress, gardening intentions, and associated mental health and physical health regarding home gardening in the context of the COVID-19 pandemic. We evaluated these hypotheses to find out whether there are any differences in the viewpoint of people in Taiwan, Thailand, and Vietnam. First, perceived stress refers to the state that a person has increased psychological issues, such as stress and distress, due to the fear or worry of the COVID-19 outbreak’s effects^24,25^. We investigated the mental well-being of citizens using a Perceived Stress Scale (PSS-4), comprising 4 items (Table S1a–c, supplementary material) because PSS-4 is a reliable, brief, and favorable psychological instrument for the perception of stress in large surveys^61–63^. According to the PSS, its items measure the degree to which an individual’s life has been appraised as stressful, uncontrollable, and unpredictable^64^. During COVID-19 outbreaks, home gardening is a suitable solution to being close to green spaces^50,52^. On a household scale, popular gardening activities are growing flowers, vegetables, fruit trees, and many types of plant^55,65,66^. Gardening intention is reflected by how often people working in the garden^67^. Since the demand to be close to green spaces still exists during the pandemic period to maintain daily lifestyle and health^45,68^, we hypothesized that perceived stress positively affects home gardening intentions (H1).

Home gardeners may face common challenges, such as being confused about suitable plants for the garden, lacking knowledge about plant care (e.g., using fertilizer and pesticide), how to use gardening tools, and how to choose the growing media^69,70^. People can be discouraged from gardening when struggling with these challenges. Thus, we developed the second hypothesis that challenges negatively affect home gardening (H2). However, there are some potential solutions to solve the challenges such as internet searching (e.g., Google, YouTube), asking experts, asking family members and friends, and taking an online class during the COVID-19 pandemic^71,72^. Those solutions support home gardeners in handling challenges. Thus, we developed the third hypothesis that solutions positively affect home gardening (H3).

**Figure 1:**
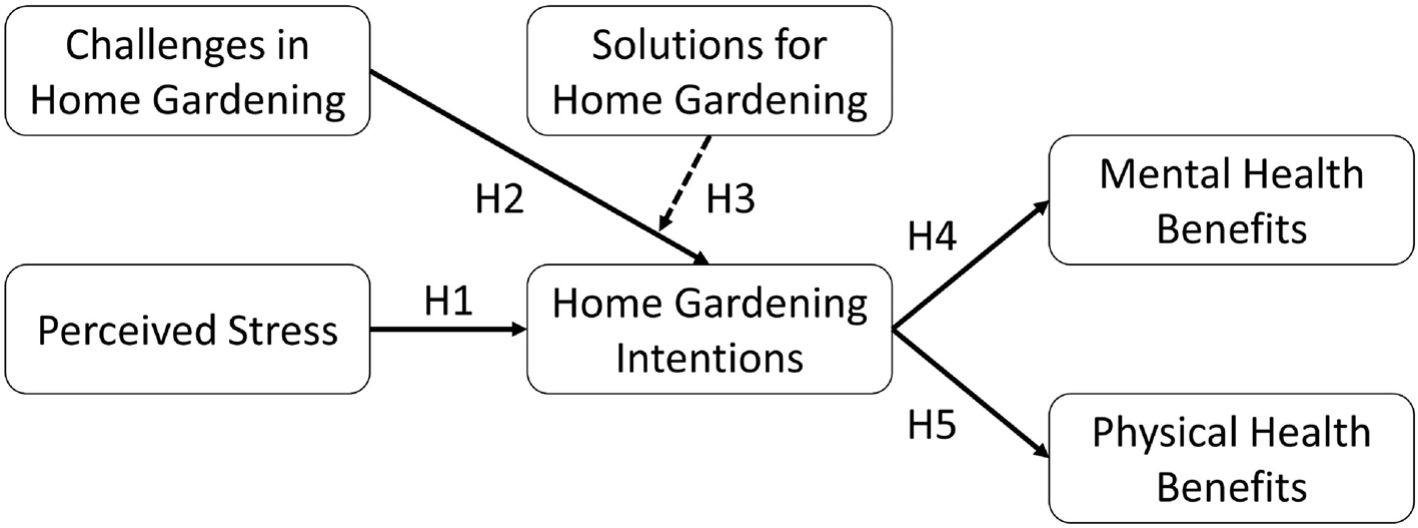
The proposed conceptual framework.

Home gardening provides multiple benefits to human mental health, such as relieving anxiety and depression^73^, decreasing loneliness^74^, stress relief^75^, and achieving relaxation^50^. Also, people can get multiple benefits for physical health from spending time working in their home gardens, such as the improvement of mobility and flexibility^76^, boosting energy^75^, muscle strength^77^, and sleep quality^78^. We hypothesized that gardening intentions positively affect mental health (H4); gardening intentions positively affect physical health (H5) during the COVID-19 pandemic. We proposed a framework, as shown in Fig. 1, where continuous arrows denote the direct effects, while dotted arrows denote the moderating effects.

## Results

### Descriptive statistics and comparisons

Details of surveys with participants’ characteristics are provided in Table S2 in the supplementary material. Regarding the comparison of participants’ personal backgrounds, respondents in Taiwan and Thailand showed more gardening experience than those in Vietnam, and the difference is statistically significant (Table S3, supplementary material). In contrast, weekly free time for gardening in participants in three countries was not significantly different at the 95% confidence interval. There is a statistically significant difference in the concerns about the COVID-19 pandemic, in which Taiwanese attendees have the highest levels of concern, followed by Thailand and Vietnam. However, Taiwanese people are less stressed than Thai and Vietnamese people during the COVID-19 pandemic. There is no significant difference between Taiwan, Thailand, and Vietnam regarding the weekly hours spent on home gardening. Respondents spend an average of 5–10 h per week gardening at home in three countries. The survey reports that Thai respondents have the highest budget for their home gardens, showing a statistically significant difference from Taiwan to Vietnam at the 95% confidence interval.

### Evaluation of measurement model

#### Internal consistency, convergent validity, and discriminant validity

In each construct, the internal consistency among variables was tested based on Cronbach’s alpha (Table S4, supplementary material). The values of Cronbach’s alpha of all constructs range from 0.764 to 0.933 (Taiwan), 0.805–0.861 (Thailand), and 0.835–0.909 (Vietnam). All values of Cronbach’s alpha come in higher than the threshold of 0.7^79,80^. The values of composite reliability of all constructs range from 0.797 to 0.958 (Taiwan), 0.824–0.969 (Thailand), and 0.839–1.553 (Vietnam), being higher than the threshold of 0.7^80,81^. The results assure the requirements of reliability in the internal consistency among constructs.

We considered the factor loadings and average variance extracted (AVE) to examine the validity of the convergence of the PLS-SEM measurement model. The acceptable factor loadings are suggested to be higher than 0.7^82^. Components with factor loadings range from 0.715 to 0.918 (Taiwan), 0.762–0.939 (Thailand), and 0.748–0.975 (Vietnam), indicating acceptable reliability levels. Values of the AVE were considered to examine the common variance in a construct^82^. In our measurement model, values of the AVE range from 0.578 to 0.799 (Taiwan), 0.628–0.819 (Thailand), and 0.665–0.799 (Vietnam), interpreting the acceptable values based on a recommended threshold of 0.5^82^. Alternatively, the values of variance inflation factors (VIF) for all items in constructs do not exceed the threshold of 5.0 (Table S1a–c, supplementary material). The result indicates satisfactory reliability, while the collinearity problem was excluded^82^.

We used the Fornell–Larcker criterion to check the discriminant validity of the measurement model, determining what extent of difference in a construct from other constructs^81^. All square roots of AVE were calculated and compared to variable correlations between constructs. The square root of AVE of each construct were higher than all other correlation values (Table S5a–c, supplementary material). Thus, the result satisfied the requirements to evaluate the discriminant validity^81^. Overall, the evaluation of our measurement model satisfied the requirements regarding internal consistency, convergent validity, and discriminant validity, interpreting the suitability of our measurement model.

#### Evaluation of path relationships

**Figure 2:**
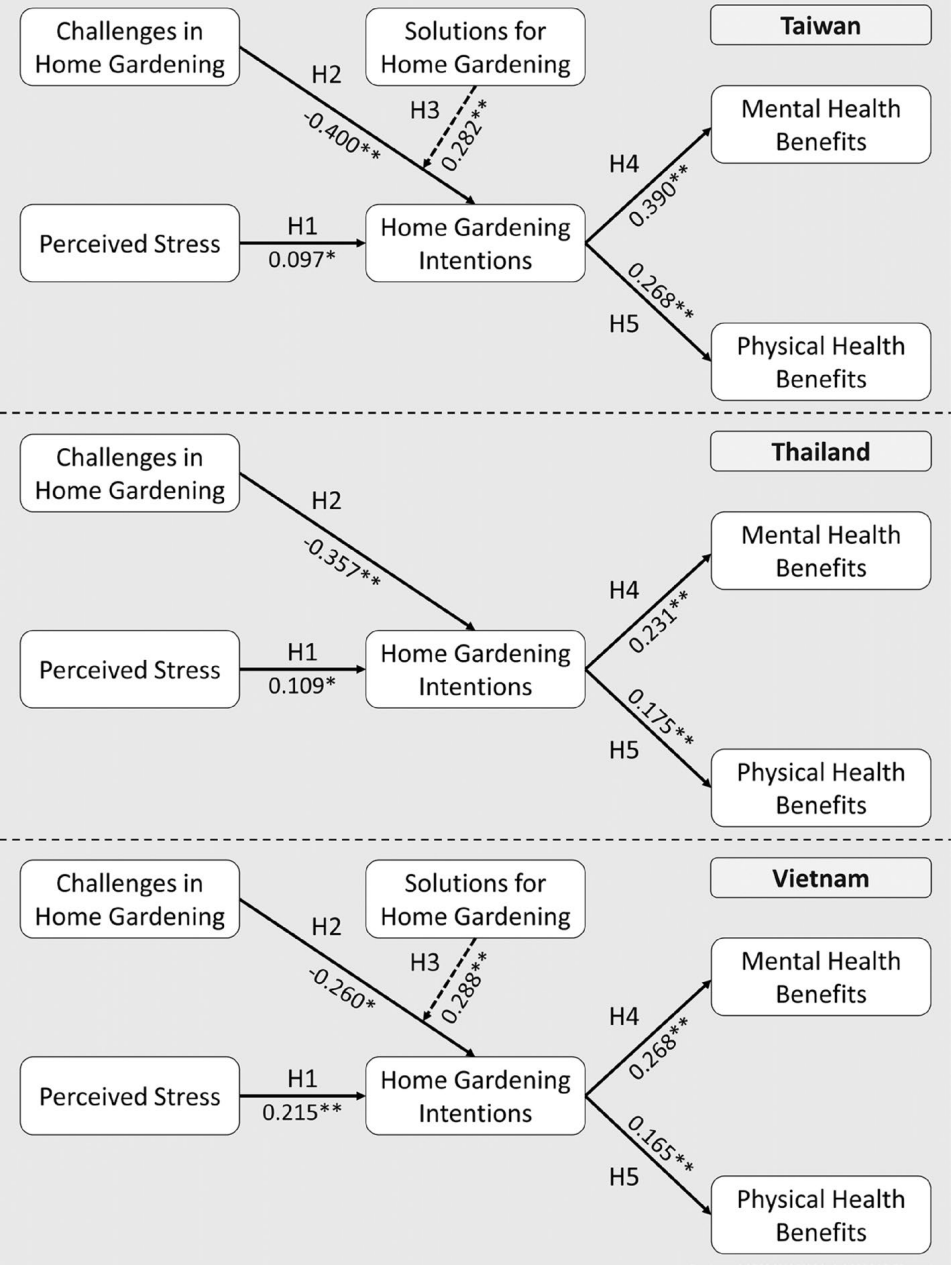
The results of PLS-SEM analysis in Taiwan, Thailand, and Vietnam.

Table 1 shows most of the proposed hypotheses are supported according to the direct relationships between constructs, with t values being higher than 1.96 at the 5% significance level, with the exception of hypothesis H3 on the relationship between solutions and home gardening in Thailand (Fig. 2). The results present that home gardening intentions are positively affected by factors such as perceived pandemic stress (βPPS → HGI = 0.097, t = 0.097, *p* = 0.097) in Taiwan, (βPPS → HGI = 0.109, t = 3.015, *p* = 0.003) in Thailand, and (βPPS → HGI = 0.215, t = 4.465, *p* =  < 0.001) in Vietnam. Solutions for home gardening also positively influenced home gardening intentions in Taiwan (βSHG → HGI = 0.282, t = 6.905, *p* =  < 0.001) and Vietnam (βSHG → HGI = 0.288, t = 3.667, *p* =  < 0.001); however, the solutions do not have an effect on home gardening in Thailand (βSHG → HGI = 0.079, t = 1.518, *p* = 0.129). On the other hand, challenges during the COVID-19 pandemic have shown negative effects on home gardening intentions in all countries (Taiwan, βCHG → HGI = − 0.4, t = 9.113, *p* =  < 0.001; Thailand, βCHG → HGI = − 0.357, t = 7.765, *p* =  < 0.001; Vietnam, βCHG → HGI = − 0.26, t = 2.346, *p* = 0.019).

**Table 1:** The results of direct effects among constructs.

	Path coefficient	Standard deviation	t value	*p* value	Result
Taiwan					
PPS → HGI	0.097	0.045	2.149	0.032	Supported H1
CHG → HGI	− 0.4	0.044	9.113	< 0.001	Supported H2
SHG → HGI	0.282	0.041	6.905	< 0.001	Supported H3
HGI → MHB	0.39	0.04	9.837	< 0.001	Supported H4
HGI → PHB	0.268	0.043	6.273	< 0.001	Supported H5
Thailand					
PPS → HGI	0.109	0.036	3.015	0.003	Supported H1
CHG → HGI	− 0.357	0.046	7.765	< 0.001	Supported H2
SHG → HGI	0.079	0.052	1.518	0.129	Rejected H3
HGI → MHB	0.231	0.042	5.53	< 0.001	Supported H4
HGI → PHB	0.175	0.042	4.186	< 0.001	Supported H5
Vietnam					
PPS → HGI	0.215	0.048	4.465	< 0.001	Supported H1
CHG → HGI	− 0.26	0.111	2.346	0.019	Supported H2
SHG → HGI	0.288	0.078	3.667	< 0.001	Supported H3
HGI → MHB	0.268	0.049	5.413	< 0.001	Supported H4
HGI → PHB	0.165	0.046	3.603	< 0.001	Supported H5

PPS, Perceived pandemic stress; HGI, Home gardening intentions; CHG, Challenges in home gardening; SHG, Solutions for home gardening; MHB, Mental health benefits; PHB, Physical health benefits.

Our results showed that home gardening intentions positively affect the benefits of mental health in all countries (Taiwan, βHGI → MHB = 0.39, t = 9.837, *p* =  < 0.001; Thailand, βHGI → MHB = 0.231, t = 5.53, *p* =  < 0.001; Vietnam, βCHG → HGI = 0.268, t = 5.413, *p* =  < 0.001). Lastly, we found that home gardening intentions also have positive effects on physical health benefits in Taiwan (βHGI → PHB = 0.268, t = 6.273, *p* =  < 0.001), Thailand (βHGI → PHB = 0.175, t = 4.186, *p* =  < 0.001), and Vietnam (βHGI → PHB = 0.165, t = 3.603, *p* =  < 0.001).

#### Moderating effects of countries—PLS-MGA

Table 2 presents the results of PLS-MGA. According to Henseler et al. (2009), the results of group A are higher than group B at a *p* value lower than 0.05. In contrast, a *p* value above 0.95 presents the result of group B being higher than group A^83^. Our results show that there is a significant difference in each pairwise comparison. Firstly, the impact of home gardening intentions on mental health benefits among Taiwanese people is higher than that among Thai people. Secondly, the impact of perceived pandemic stress on home gardening intentions among Vietnamese surveyed attendees is higher than that among Taiwanese and Thai participants. Finally, we found that there are non-significant differences across groups for other relationships across Taiwan, Thailand, and Vietnam.

**Table 2:** The results of multigroup analysis.

Groups	Path coefficient difference	*p* value (one-tailed)
Taiwan—Thailand		
PPS → HGI	− 0.012	0.580
CHG → HGI	− 0.043	0.749
HGI → MHB	0.159	0.003*
HGI → PHB	0.094	0.059
Taiwan—Vietnam		
PPS → HGI	− 0.118	0.970*
CHG → HGI	− 0.140	0.928
SHG → HGI	− 0.006	0.542
HGI → MHB	0.122	0.027
HGI → PHB	0.103	0.050
Thailand—Vietnam		
PPS → HGI	− 0.106	0.963*
CHG → HGI	− 0.097	0.816
HGI → MHB	− 0.037	0.715
HGI → PHB	0.009	0.442

PPS, Perceived pandemic stress; HGI, Home gardening intentions; CHG, Challenges in home gardening; SHG, Solutions for home gardening; MHB, Mental health benefits; PHB, Physical health benefits.

*denotes *p* value < 0.05 or *p* value > 0.95.

## Discussion

### Theoretical implications

Several studies have investigated how being close to nature improves health conditions^41,43,84^. However, understanding the linkages among perceived stress, green spaces, and health benefits still needs to be improved in the scientific literature. Our study developed a conceptual model to examine why people do home gardening and how home gardening affects physical and mental well-being in the context of the COVID-19 pandemic. In the present study, perceived pandemic stress, challenges in gardening, and solutions were organized to be antecedents to home gardening intentions toward mental and physical health benefits in Taiwan, Thailand, and Vietnam. Regarding determinants affecting the intentions of home gardening, we found that perceived stress positively affects people doing home gardening during the pandemic in three countries. Our result provided further evidence of the core demand of being close to nature during the enforced lockdown and social distancing periods^45,68^. The path coefficient of PPS → HGI for Vietnam presents the highest value and significantly differs from Thailand to Taiwan, while Vietnamese respondents also indicated their higher stress levels than people in Taiwan and Thailand (Table S3, supplementary material). The differences could be caused by how people experienced the impacts of the spread of COVID-19 across three countries, whereby Vietnam was experiencing a surge in the highest total of confirmed cases and deaths since the start of the pandemic among three countries^1,10^. Our result is in line with those from previous studies in the US and China, indicating that persistent psychological and health problems further increase the demands for nature connections to relieve human stress and boost well-being^45,50^.

Challenges such as lacking knowledge about plant care and using gardening tools were the typical factors that hindered the intentions of home gardening of surveyed attendees in Taiwan, Thailand, and Vietnam. Some previous studies have indicated that beginners encounter these challenges in home gardening due to their lack of experience^85^. This is consistent with the results in our research that respondents are the majority aged less than 45 and more than 40% of those with less than 1 year of gardening experience. Therefore, it is unsurprising that a lack of gardening knowledge and skills hinders their intention to garden at home. We also proposed potential solutions for overcoming the challenges in home gardening, which are suitable in the context of the COVID-19 pandemic. The solutions have shown positive impacts on the home gardening intentions of the respondents in Taiwan and Vietnam. The results are in line with previous studies indicating online gardening courses can advance people’s knowledge of horticultural activities and promote the participant’s confidence to do gardening^72^. Consulting with experts or self-learning about growing plants online can also solve fundamental gardening problems^71^, particularly in complying with social distancing during the pandemic.

While the availability of online resources, including websites and courses, can help people learn about gardening, the finding of Thai respondents does not coincide with Taiwan and Vietnam. The social norms surrounding plant care varying between three countries may influence people’s behaviors in choosing different solutions to solve the challenges in home gardening during COVID-19 pandemic^86^. To our knowledge, it is common for Thai people to get personalized advice and information that is tailored to the specific plant they are interested in by asking sellers directly about plant care at markets like Chatuchak Market in Bangkok, Thailand. Accordingly, Thai people believe it is convenient to ask many plant sellers with extensive knowledge and experience with the plants they sell rather than researching on their own. However, the closure of markets as a precautionary measure because of COVID-19 worries may make it challenging for Thai people to care for their home gardens as they cannot interact directly and seek advice from plant sellers. The availability of online support tools, such as online gardening classes, remains a need in Thailand during the COVID-19 pandemic^87^; thus, it may become a barrier for individuals to find plant care solutions. Meanwhile, Wu et al. 2022 have demonstrated the inclination of individuals to take part in online gardening classes in Taiwan to reduce their COVID-19-related stress. On the other hand, searching online for information on plant care has long been popular among Vietnamese urban gardeners^88^. Hence, it may be necessary to consider alternative solutions in future studies to support individuals engaging in home gardening in Thailand.

Most respondents were young people, with an average age under 45 years old; therefore, the result was not surprising that the ratio of participants with an average gardening experience below 5 years was relatively high, whereby Vietnamese have the least experience. On the other hand, there is no significant difference in the hours per week people in all countries spend on home gardening. Our result is consistent with a study by Egerer et al. (2022), showing that people with less than 10 years of gardening experience spend similar time working in gardens. Our study indicates that home gardening intentions positively impact physical and mental health benefits among surveyed attendees in Taiwan, Thailand, and Vietnam. The result is consistent with the findings from recent studies in Taiwan, Singapore, and the US that home gardening benefits human health^56,75,89^. However, there is a significant difference between Taiwan and Thailand regarding the path coefficient of home gardening intentions on mental health benefits (HGI → MHB). While both countries have been affected by COVID-19, Thailand has suffered greater severity of the virus’s impact than Taiwan regarding the deaths^1,10^. In fact, people in Thailand perceived more stress than those in Taiwan (Table S3, supplementary material). Yet the duration of time dedicated to gardening is similar, leading to a lower path coefficient of home gardening in promoting mental health benefits in Thailand than in Taiwan.

Our findings contribute to the theoretical implications of providing a better understanding of how people in these countries do home gardening and the associated benefits that they obtained during the pandemic outbreaks. In conclusion, perceived pandemic stress promotes home gardening among people in Taiwan, Thailand, and Vietnam, while challenges in gardening knowledge and skills negatively impact the intentions of gardening at home. However, the solutions supporting home gardening are only effective in Taiwan and Vietnam. Our results show that home gardening benefits mental and physical health in Taiwan, Thailand, and Vietnam. We also employed the multigroup analysis to examine the impact of different countries on the relationships between constructs in our conceptual model. We found that Thai people have lower home gardening intentions for mental health benefits than Taiwanese. Finally, Vietnamese people have higher perceived pandemic stress for home gardening intentions than other Taiwanese and Thai people.

The current study is not without limitations. Regarding the sample collection, most respondents in Taiwan, Thailand, and Vietnam were aged under 65, while the portion of people over 65 years old only accounts for less than 7%. We understand that the convenience sampling method cannot avoid the different proportions of age groups in the sample. However, we encourage future studies focusing on the elderly age group because this is a vulnerable age group to the COVID-19 pandemic^90,91^. Similar efforts are needed to find the benefits of home gardening to protect older populations in different countries. Despite the health benefits being important, gardening at home also provides other benefits in terms of food and social aspects^55,92^. Therefore, future studies should consider these two aspects to further strengthen the theory of the benefits of home gardening during the COVID-19 pandemic.

## Research strengths and limitations

In our research, using questionnaires to evaluate the benefits of home gardening on physical and mental health presents both strengths and limitations. The questionnaire-based survey offers the friendly accessibility to encourage citizen participation in Taiwan, Thailand, and Vietnam. To comply with social distancing regulations in these countries, using an online questionnaire offers the convenience to acquire samples that resolve the difficulty associated with face-to-face interactions during COVID-19 pandemic. Thus, the process renders data collection from diverse samples across populations in Taiwan, Thailand, and Vietnam conductively and possibly. Moreover, the longitudinal nature of our survey allows future studies to investigate and capture the trends in people’s perceptions of home gardening benefits at multiple time points of the COVID-19 pandemic, particularly among vulnerable population segments^51,93^.

On the other hand, our study has limitations that need to be addressed for future research. The research based on questionnaires collected subjective answers that may be susceptible to inaccuracies in participants’ recollections^94,95^. Accordingly, potential discrepancies between participants’ past experiences on their health outcomes of home gardening and their responses to the questionnaire may occur. Additionally, our questionnaires did not obtain or analyze the physiological or neurological data that may not present the specific mechanisms underlying the effects of home gardening. Future research can employ brain scanners such as functional near-infrared spectroscopy (fMRI) and multimodal electroencephalography (EEG), along with the Profile of Mood States (POMS) questionnaire^42^ to gain a deeper understanding of neural mechanisms of the therapeutic benefits of home gardening to human health. For instance, brain scanning techniques allow researchers to establish experimental designs to determine the cause-and-effect relationships between home gardening and mental well-being. The approach enables comparisons between clinical groups, such as individuals suffering from depression, and non-clinical groups, thereby generalizing the neural mechanisms underlying the impacts of home gardening on the mental health benefits across different populations.

## Methods

### Social survey

We conducted a questionnaire-based survey to collect data for the purpose of this study. We established a research team among professors from, National Chung Hsing University in Taiwan, Kasetsart University in Thailand, and Nong Lam University—Ho Chi Minh City in Vietnam, to conduct surveys three universities. To ensure a comparable number of participants, we used Google Forms to design the language-specific questionnaires in Chinese, Thai, and Vietnamese to engage participants in their mother tongues. In each country, we conducted a cross-sectional survey by posting questionaries on a social media platform (Facebook) to collect data by convenience sampling method^96^ from May 1 to September 30 in 2022. We also shared the questionaries within specific Facebook pages and groups related to home gardening to attract more targeted participants (i.e., home gardeners) engaging the survey. Our Facebook posts were shareable to facilitate snowball sampling^97^, encouraging people to distribute the questionnaire to their friends and relatives. Our survey questionnaire contains 24 variables (Table S1a–c, supplementary material). Thus, we estimated a minimum sample size of 240 participants for each country according to the 10-times rule to obtain reliable data^82,98^.

There are three main sections of the questionnaire. In the first section, we clarified the purposes of our study and showed brief instructions on how to answer the questionnaire. In the second section, we collected basic personal information, including gender, age, gardening experience, weekly free time, levels of concern, levels of stress, weekly hours in home gardening, and home garden budget. The last section included questions regarding perceived pandemic stress (PPS), home gardening intentions (HGI), challenges in home gardening (CHG), solutions for home gardening (SHG), mental health benefits (MHB), and physical health benefits (PHB) (Table S1a–c, supplementary material). For PPS and HGI, a 5-point Likert scale was used to rate each item, ranging from 1 (i.e., never) to 5 (i.e., very often). We measured items regarding CHG, SHG, MHB, and PHB using 6 point-Likert scale questions (1: Strongly disagree and 6: Strongly agree).

We collected and analyzed anonymous data from research participants in Taiwan without conducting biomedical or medical experiments involving human subjects. Therefore, our study falls under the “Human research” category, which does not require review by a research ethics board according to the Taiwan Human Subjects Research Act^99,100^. In Thailand and Vietnam, the regulatory framework stipulates that research involving human participants without biomedical or medical experiments (e.g., social surveys) does not require ethical approval from an institutional ethics committee prior to conducting the study^101,102^. Therefore, the present research and associated protocols have been determined to fall outside the scope of ethical approval by any institutional ethics committee in accordance with the regulatory requirements of the host countries. In this survey, we ensured that all participants had been duly informed of the purpose of the study and provided with the autonomy to either continue or decline participation. All respondents answer the questionnaire anonymously. We complied with guidelines for human ethics that participants were provided informed consent and assured of confidentiality. We did not collect personally identifiable data, and there were no observations, interventions, or interactions. Participants agreed to participate in the survey and accomplished the questionnaires, which were considered valid responses when all questions were completely answered. We finally collected the samples (N) consisting of 374, 448, and 350 valid responses from Taiwan, Thailand, and Vietnam, respectively.

### Statistical analysis

This study tested the conceptual model and the hypotheses using the Partial Least Square Structural Equation Modeling (PLS-SEM). PLS-SEM is developed based on a least squares-based method, an advanced approach to covariance-based structural equation modeling (CB-SEM)^103^. PLS-SEM has received academic favorability in recent studies to analyze the data in lieu of CB-SEM. PLS-SEM enables researchers to test the complexity of the relationships between latent variables in many constructs^104^. Following the research objectives, we first considered the measurement model following 3 main criteria: internal consistency, convergent validity, and discriminant validity to evaluate the reliability of the measurement model^103^. Then, we assessed the structural model to explain the relationship between latent variables in each country. The direct relationships between constructs were examined using path coefficients (β). We run bootstrapping analysis with 5000 resamples to examine the significant differences in path coefficients among constructs based on the *p* value and t value. A path relationship is deemed to be significant with a *p* value less than 0.05 and a t value higher than 1.96^82^. In the final step, we conducted a Partial Least Squares-Multigroup Analysis (PLS-MGA), a non-parametric significance test, to understand the difference between countries (i.e., groups), such as Taiwan–Thailand, Taiwan–Vietnam, and Thailand–Vietnam, towards constructs. A 5000 bootstrap-based MGA approach was used to demonstrate significant differences between path coefficients among groups at a 5% probability of error level, where the *p* value was greater than 95% or lower than 5%, denoting the certain difference in group-specific path coefficients^83^. We used SmartPLS 4.0 software to perform the PLS-SEM and PLS-MGA^105,106^.

## Supplementary Information

Supplementary Information.

## Data Availability

The datasets used and/or analysed during the current study available from the corresponding author on reasonable request.
