# Supplementary material for: Enhancing human resilience beyond COVID-19-related stress: public responses to multi-benefits of home gardening
Source: Sci Rep. 2023 Jun 29;13:10534. doi: 10.1038/s41598-023-37426-0 (PMC10310725; doi:10.1038/s41598-023-37426-0)
Supplement: Supplementary file 1 — Supplementary Information. [file 41598_2023_37426_MOESM1_ESM.pdf]

Enhancing human resilience beyond COVID-19-related stress: Public responses to multi-benefits of home gardening

Chen-Fa Wu [a, b], Luu Van Thong Trac [a, *], Szu-Hung Chen [c, *], Alisara Menakanit [d], Quoc Tuan Le [e], Hung-Ming Tu [a], Chih-Peng Tsou [a], Hsi-Chih Huang [a], Nittaya Chookoh [d], Chih-Cheng Weng [f], Li-Wei Chou [g, h, i], Chiu-Chuan Chen [j]

[a] Department of Horticulture, National Chung Hsing University, Taichung City 402, Taiwan

[b] Innovation and Development Center of Sustainable Agriculture, National Chung Hsing University, Taichung City 402, Taiwan

[c] International Master Program of Agriculture, National Chung Hsing University, Taichung City 402, Taiwan

[d] Department of Horticulture, Kasetsart University, Bangkok 10900, Thailand

[e] Department of Environmental Sciences, Faculty of Environment and Natural Resources, Nong Lam University - Ho Chi Minh City, Ho Chi Minh City 700000, Vietnam

[f] Miaoli Management Office, Irrigation Agency, Council of Agriculture, Executive Yuan, Miaoli County 360, Taiwan

[g] Department of Physical Medicine and Rehabilitation, China Medical University Hospital, 404332, Taichung, Taiwan

[h] Department of Physical Therapy and Graduate Institute of Rehabilitation Science, China Medical University, 406040, Taichung, Taiwan

[i] Department of physical Medicine and Rehabilitation, Asia University Hospital, Asia University, 413505, Taichung, Taiwan

[j] Department of Landscape Architecture, Chung Chou University of Science and Technology, Changhua County 510, Taiwan

Contact details

Dr. Chen-Fa Wu; cfwu@dragon.nchu.edu.tw

Ph.D. student Luu Van Thong Trac; thongtracluuvan@smail.nchu.edu.tw

Dr. Szu-Hung Chen; vickey@dragon.nchu.edu.tw

Dr. Alisara Menakanit; agrarm@ku.ac.th

Dr. Quoc Tuan Le; quoctuan@hcmuaf.edu.vn

Dr. Hung-Ming Tu; hmtu@dragon.nchu.edu.tw

Ph.D. student Chih-Peng Tsou; d110032004@mail.nchu.edu.tw

Dr. Hsi-Chih Huang; m0605338@mail.fcu.edu.tw

Dr. Nittaya Chookoh; nittaya.cho@ku.th

Dr. Chih-Cheng Weng; wengcc@nchu.edu.tw

Dr. Li-Wei Chou; chouliwe@mail.cmu.edu.tw

Dr. Chiu-Chuan Chen; land530308@gmail.com

*** Corresponding authors**

Ph.D. student Luu Van Thong Trac

Tel: +886-970-689-579; Email: thongtracluuvan@smail.nchu.edu.tw

Department of Horticulture, National Chung Hsing University, Taichung City 402, Taiwan

Dr. Szu-Hung Chen

Tel: +886-4-2285-9125; Email: vickey@dragon.nchu.edu.tw

International Master Program of Agriculture, National Chung Hsing University, Taichung City 402, Taiwan

Abbreviation

PPS: Perceived pandemic stress

HGI: Home gardening intentions

CHG: Challenges in home gardening

SHG: Solutions for home gardening

MHB: Mental health benefits

PHB: Physical health benefits

Table S1a. The items of the measurement model of Taiwan

Variable	Measurement scales	Original sample	Sample mean	Standard deviation	VIF
**Perceived Pandemic Stress (PPS)**					
PPS1	How often have you felt that you were unable to control the important things in your life?	0.715	0.672	0.156	1.536
PPS2	How often have you felt confident about your ability to handle your personal problems?	0.816	0.784	0.131	1.454
PPS3	How often have you felt that things were going your way?	0.783	0.752	0.126	1.467
PPS4	How often have you felt difficulties were piling up so high that you could not overcome them?	0.722	0.683	0.147	1.428
**Home Gardening Intentions (HGI)**					
HGI1	Growing flowers	0.840	0.840	0.021	1.995
HGI2	Growing vegetables	0.887	0.886	0.016	2.712
HGI3	Growing fruit trees	0.780	0.778	0.033	1.802
HGI4	Growing many types of plant	0.775	0.775	0.029	1.571
**Challenges in home gardening (CHG)**					
CHG1	I am confused about suitable plants for the garden.	0.854	0.853	0.030	1.838
CHG2	I have lacking knowledge about plant care (e.g., using fertilizer and pesticides).	0.913	0.912	0.014	2.727
CHG3	I do not how to use gardening tools and how to choose the growing media.	0.860	0.859	0.027	2.168
**Solutions for home gardening (SHG)**					
SHG1	Internet searching (e.g., Google, YouTube)	0.789	0.786	0.052	1.606
SHG2	Asking experts	0.809	0.794	0.083	4.118
SHG3	Asking family members and friends	0.758	0.738	0.107	3.892
SHG4	Taking an online gardening course	0.793	0.790	0.060	1.525
**Mental health benefits (MHB)**					
MHB1	Relieving anxiety	0.873	0.874	0.016	2.614
MHB2	Reducing depression	0.910	0.910	0.013	3.780
MHB3	Decreasing loneliness	0.909	0.908	0.014	4.127
MHB4	Stress relief	0.889	0.888	0.019	4.154
MHB5	Achieving relaxation	0.852	0.850	0.030	3.143
**Physical health benefits (PHB)**					
PHB1	Improving mobility and flexibility	0.880	0.881	0.018	2.208
PHB2	Boosting energy	0.918	0.917	0.012	3.648
PHB3	Improving muscle strength	0.887	0.884	0.022	3.621
PHB4	Improving sleep quality	0.890	0.887	0.023	3.629

Table S1b. The items of the measurement model of Thailand

Variable	Measurement scales	Original sample	Sample mean	Standard deviation	VIF
**Perceived Pandemic Stress (PPS)**					
PPS1	How often have you felt that you were unable to control the important things in your life?	0.822	0.806	0.092	1.978
PPS2	How often have you felt confident about your ability to handle your personal problems?	0.762	0.740	0.112	1.922
PPS3	How often have you felt that things were going your way?	0.780	0.755	0.111	1.792
PPS4	How often have you felt difficulties were piling up so high that you could not overcome them?	0.805	0.788	0.102	1.773
**Home Gardening Intentions (HGI)**					
HGI1	Growing flowers	0.872	0.872	0.020	2.471
HGI2	Growing vegetables	0.832	0.833	0.019	2.001
HGI3	Growing fruit trees	0.841	0.840	0.021	2.037
HGI4	Growing many types of plant	0.776	0.775	0.026	1.565
**Challenges in home gardening (CHG)**					
CHG1	I am confused about suitable plants for the garden.	0.897	0.896	0.019	2.283
CHG2	I have lacking knowledge about plant care (e.g., using fertilizer and pesticides).	0.845	0.844	0.025	1.841
CHG3	I do not how to use gardening tools and how to choose the growing media.	0.886	0.886	0.015	2.196
**Solutions for home gardening (SHG)**					
SHG1	Internet searching (e.g., Google, YouTube)	0.888	0.890	0.020	2.431
SHG2	Asking experts	0.852	0.848	0.033	2.500
SHG3	Asking family members and friends	0.782	0.777	0.048	1.785
SHG4	Taking an online gardening course	0.832	0.828	0.032	2.110
**Mental health benefits (MHB)**					
MHB1	Relieving anxiety	0.833	0.835	0.027	2.111
MHB2	Reducing depression	0.911	0.908	0.014	4.143
MHB3	Decreasing loneliness	0.862	0.859	0.025	3.358
MHB4	Stress relief	0.876	0.873	0.023	4.146
MHB5	Achieving relaxation	0.860	0.858	0.023	3.173
**Physical health benefits (PHB)**					
PHB1	Improving mobility and flexibility	0.857	0.855	0.027	2.748
PHB2	Boosting energy	0.939	0.938	0.010	4.494
PHB3	Improving muscle strength	0.935	0.935	0.014	4.404
PHB4	Improving sleep quality	0.887	0.884	0.028	3.764

Table S1c. The items of the measurement model of Vietnam

Variable	Measurement scales	Original sample	Sample mean	Standard deviation	VIF
**Perceived Pandemic Stress (PPS)**					
PPS1	How often have you felt that you were unable to control the important things in your life?	0.826	0.822	0.031	1.906
PPS2	How often have you felt confident about your ability to handle your personal problems?	0.832	0.835	0.030	1.601
PPS3	How often have you felt that things were going your way?	0.809	0.802	0.048	1.919
PPS4	How often have you felt difficulties were piling up so high that you could not overcome them?	0.793	0.785	0.047	1.878
**Home Gardening Intentions (HGI)**					
HGI1	Growing flowers	0.808	0.808	0.026	1.764
HGI2	Growing vegetables	0.793	0.791	0.032	1.842
HGI3	Growing fruit trees	0.844	0.843	0.021	2.200
HGI4	Growing many types of plant	0.826	0.826	0.028	2.073
**Challenges in home gardening (CHG)**					
CHG1	I am confused about suitable plants for the garden.	0.823	0.754	0.247	2.180
CHG2	I have lacking knowledge about plant care (e.g., using fertilizer and pesticides).	0.975	0.883	0.224	3.188
CHG3	I do not how to use gardening tools and how to choose the growing media.	0.878	0.797	0.235	2.893
**Solutions for home gardening (SHG)**					
SHG1	Internet searching (e.g., Google, YouTube)	0.898	0.883	0.081	3.583
SHG2	Asking experts	0.863	0.854	0.080	2.509
SHG3	Asking family members and friends	0.905	0.898	0.071	3.126
SHG4	Taking an online gardening course	0.875	0.867	0.079	2.612
**Mental health benefits (MHB)**					
MHB1	Relieving anxiety	0.904	0.903	0.019	3.860
MHB2	Reducing depression	0.748	0.744	0.043	2.317
MHB3	Decreasing loneliness	0.897	0.895	0.021	3.876
MHB4	Stress relief	0.770	0.766	0.043	2.433
MHB5	Achieving relaxation	0.903	0.902	0.017	3.621
**Physical health benefits (PHB)**					
PHB1	Improving mobility and flexibility	0.833	0.829	0.047	1.953
PHB2	Boosting energy	0.820	0.811	0.057	2.093
PHB3	Improving muscle strength	0.853	0.846	0.044	2.435
PHB4	Improving sleep quality	0.848	0.844	0.042	2.287

Descriptive statistics of respondents in Taiwan, Thailand, and Vietnam

The total number of residents who participated in our study in Taiwan was 153 males (40.9%) and 221 females (59.15), while 134 males (29.9%) and 314 females (70.1%) participated in the survey in Thai. In Vietnam, 138 males and 212 females participated in the survey, accounting for 39.4% and 60.6%, respectively. The age group from 21 to 45 years old accounts for the highest percentage of participants in the three countries (over 40%), while the age group less than 20 years old accounts for 13.4% to 29.1%, followed by age group from 46 to 65 years old with the percentage ranging from 17.7 to 27.0. For the older age group, participants over 65 accounts for the minor proportions, with 4.0%, 6.3, and 1.4% in Taiwan, Thailand, and Vietnam, respectively.

The proportion of participants with experience in gardening less than 5 years was relatively high in Taiwan (71.1%), Thailand (71.2), and Vietnam (82%), while participants with over 6 years experienced in gardening accounted for 29%, 28.8%, and 17.9% in Taiwan, Thailand, and Vietnam, respectively. Most survey attendees are working age population (i.e., those aged from 15 to 64); thus, the total participants who have less than 1 day and from 1 to 2 free days every week account for the largest portions in Taiwan (70.3%), Thailand (75.2%), and Vietnam (67.7%), whereas people with 2 and 3 free days per week account for 20.3%, 13.8%, and 16.3% in Taiwan, Thailand, and Vietnam, respectively. In contrast, there are minor percentages of 9.3 % (Taiwan), 10.8% (Thailand), and 16% (Vietnam) people with weekly free days from 3 to 7 accounting.

Most of the participants in the countries have high levels of concern about the COVID-19 pandemic, from slightly concerned to totally concerned, accounting for 74.5%, 73.5%, and 52.6% in Taiwan, Thailand, and Vietnam, respectively, whereby Taiwan has the highest percentage of people who totally concerned with 23.5%. In contrast, 22.3% of people surveyed in Vietnam showed slight unconcern about the COVID-19 pandemic, while the proportion in Taiwan and Thailand was 15.2% and 17.0%, respectively. The total percentage of people who are unconcern and totally unconcerned is also highest in Vietnam, accounting for 25.2%, followed by Thailand (9.6%) and Taiwan (10.1%). Regarding stress levels, most Vietnamese survey attendees are often and always stressed during the COVID-19 pandemic, accounting for 43.7%, while 31.7% of people sometimes have stress during this period. In Taiwan and Thailand, 27.8% and 34.6% of participants are often and always stressed, followed by 31.3% and 40.25% of people who are sometimes stressed during the COVID-19 pandemic, respectively. Taiwanese survey attendees who were rarely and never stressed during the pandemic accounted for the highest proportion (40.9%) in three countries, while the lower rates are witnessed in Thailand (25.2%) and Vietnam (24.5%).

More than 45% of people surveyed in each country indicated that they spend 1 to 5 hours a week on gardening, followed by 24.9%, 28.3%, and 19.4% of participants in Taiwan, Thailand, and Vietnam spend less than 1 hour weekly on gardening. From 11.55% to 16.0% of surveyed attendees spend 5 to 10 hours a week on gardening. The smallest proportion is those who spend more than 10 hours gardening per week, with 9.3%, 13.8%, and 12.9% in Taiwan, Thailand, and Vietnam, respectively. Those surveyed with a home gardening budget of less than 15 USD account for 17.1% in Taiwan, 17.2% in Thailand, and 29.4% in Vietnam, while 77.1% of Taiwanese, 69.2% of Thais, and 59.7% of Vietnamese showed they are willing to pay between 15 USD and 80 USD for their home gardens. The smallest proportion can be seen in those with a budget of more than 400 USD for home gardening, with 6.0%, 1.4%, and 3.4% in Taiwan, Thailand, and Vietnam, respectively.

Table S2. The descriptive statistics of respondent characteristics.

		Taiwan		Thailand		Vietnam	
		N	%	N	%	N	%
Gender	Male	153	40.9	134	29.9	138	39.4
	Female	221	59.1	314	70.1	212	60.6
	Total	374	100.0	448	100.0	350	100.0
Age	Less than 20 years old	50	13.4	111	24.8	102	29.1
	From 21 to 45 years old	208	55.6	188	42.0	181	51.7
	From 46 to 65 years old	101	27.0	121	27.0	62	17.7
	Over 65 years old	15	4.0	28	6.3	5	1.4
	Total	374	100.0	448	100.0	350	100.0
Gardening experience	No experience	60	16.0	95	21.2	138	39.4
	Less than 1 year	99	26.5	120	26.8	77	22.0
	From 1 to 5 years	107	28.6	104	23.2	72	20.6
	From 6 to 10 years	50	13.4	45	10.0	25	7.1
	From 11 to 20 years	26	7.0	42	9.4	19	5.4
	Over 21 years	32	8.6	42	9.4	19	5.4
	Total	374	100.0	448	100.0	350	100.0
Weekly free time	Less than 1 day	83	22.2	70	15.6	51	14.6
	From 1 to 2 days	180	48.1	267	59.6	186	53.1
	From 2 to 3 days	76	20.3	62	13.8	57	16.3
	From 3 to 4 days	11	2.9	19	4.2	26	7.4
	From 4 to 5 days	11	2.9	10	2.2	14	4.0
	From 5 to 6 days	4	1.1	2	0.4	7	2.0
	From 6 to 7 days	9	2.4	18	4.0	9	2.6
	Total	374	100.0	448	100.0	350	100.0
Levels of concern	Totally unconcerned	14	3.7	15	3.3	44	12.6
	Unconcerned	24	6.4	28	6.3	44	12.6
	Slightly unconcerned	57	15.2	76	17.0	78	22.3
	Slightly concerned	91	24.3	168	37.5	86	24.6
	Concerned	100	26.7	98	21.9	62	17.7
	Totally concerned	88	23.5	63	14.1	36	10.3
	Total	374	100.0	448	100.0	350	100.0
Levels of stress	Never	37	9.9	30	6.7	18	5.1
	Rarely	116	31.0	83	18.5	68	19.4
	Sometimes	117	31.3	180	40.2	111	31.7
	Often	83	22.2	113	25.2	117	33.4
	Always	21	5.6	42	9.4	36	10.3
	Total	374	100.0	448	100.0	350	100.0
Weekly hours in home gardening	Less than 1 hour	93	24.9	127	28.3	68	19.4
	From 1 to 5 hours	203	54.3	205	45.8	181	51.7
	From 5 to 10 hours	43	11.5	54	12.1	56	16.0
	From 10 to 20 hours	14	3.7	31	6.9	24	6.9
	From 20 to 30 hours	8	2.1	29	6.5	10	2.9
	Over 30 hours	13	3.5	2	0.4	11	3.1
	Total	374	100.0	448	100.0	350	100.0
Home garden budget	Less than 15 USD	64	17.1	77	17.2	103	29.4
	From 15 to 30 USD	124	33.2	103	23.0	107	30.6
	From 30 to 50 USD	120	32.1	133	29.7	54	15.4

	Taiwan		Thailand		Vietnam	
	N	%	N	%	N	%
From 50 to 80 USD	44	11.8	74	16.5	48	13.7
From 80 to 200 USD	13	3.5	27	6.0	21	6.0
From 200 to 400 USD	3	0.8	11	2.5	5	1.4
Over 400 USD	6	1.6	23	5.1	12	3.4
Total	374	100.0	448	100.0	350	100.0

Table S3. The differences in the personal backgrounds of participants in Taiwan, Thailand, and Vietnam

Category	Country	N	Mean	SD	p-value/F-value
Gardening experience	Taiwan	374	2.94[b]	1.446	<0.001/18.269
	Thailand	448	2.88[b]	1.557	
	Vietnam	350	2.33[a]	1.462	
Weekly free time	Taiwan	374	2.29	1.224	
	Thailand	448	2.35	1.273	0.088/2.439
	Vietnam	350	2.49	1.300	
Levels of concern	Taiwan	374	4.34[c]	1.357	<0.001/34.556
	Thailand	448	4.10[b]	1.223	
	Vietnam	350	3.53[a]	1.494	
Levels of stress	Taiwan	374	2.83[a]	1.061	<0.001/15.453
	Thailand	448	3.12[b]	1.034	
	Vietnam	350	3.24[b]	1.044	
Weekly hours in home gardening	Taiwan	374	2.14	1.116	0.111/2.201
	Thailand	448	2.19	1.141	
	Vietnam	350	2.31	1.137	
Home garden budget	Taiwan	374	2.60[a]	1.203	<0.001/11.850
	Thailand	448	2.99[b]	1.540	
	Vietnam	350	2.54[a]	1.528	

Mean values in the same column with the same letter and are not significantly different (SD: standard deviation)

Table S4. The model evaluation of Taiwan, Thailand, and Vietnam

Constructs	Items	Factor loading			Cronbach's alpha			Composite Reliability			Average Variance Extracted		
		Taiwan	Thailand	Vietnam	Taiwan	Thailand	Vietnam	Taiwan	Thailand	Vietnam	Taiwan	Thailand	Vietnam
Perceived pandemic stress (PPS)					0.764	0.805	0.835	0.797	0.824	0.863	0.578	0.628	0.665
	PPS1	0.715	0.822	0.826									
	PPS2	0.816	0.762	0.832									
	PPS3	0.783	0.78	0.809									
	PPS4	0.722	0.805	0.793									
Home gardening intentions (HGI)					0.839	0.85	0.835	0.841	0.85	0.839	0.675	0.69	0.669
	HGI1	0.84	0.872	0.808									
	HGI2	0.887	0.832	0.793									
	HGI3	0.78	0.841	0.844									
	HGI4	0.775	0.776	0.826									
Challenges in home gardening (CHG)					0.848	0.848	0.889	0.849	0.852	1.553	0.767	0.768	0.799
	CHG1	0.854	0.897	0.823									
	CHG2	0.913	0.845	0.975									
	CHG3	0.86	0.886	0.878									
Solutions for home gardening (SHG)					0.802	0.861	0.909	0.817	0.902	0.923	0.62	0.704	0.784
	SHG1	0.789	0.888	0.898									
	SHG2	0.809	0.852	0.863									
	SHG3	0.758	0.782	0.905									
	SHG4	0.793	0.832	0.875									
Mental health benefits (MHB)					0.933	0.92	0.902	0.958	0.95	0.932	0.787	0.755	0.718
	MHB1	0.873	0.833	0.904									
	MHB2	0.91	0.911	0.748									
	MHB3	0.909	0.862	0.897									
	MHB4	0.889	0.876	0.77									
	MHB5	0.852	0.86	0.903									
Physical health benefits (PHB)					0.918	0.927	0.86	0.952	0.969	0.869	0.799	0.819	0.703
	PHB1	0.88	0.857	0.833									
	PHB2	0.918	0.939	0.82									
	PHB3	0.887	0.935	0.853									
	PHB4	0.89	0.887	0.848									

Table S5a. The Fornell-Larcker criterion in discriminant validity assessment (Taiwan)

	MHB	CHG	HGI	PHB	PPS	SHG
MHB	0.887					
CHG	0.096	0.876				
HGI	0.39	-0.34	0.822			
PHB	0.544	0.091	0.268	0.894		
PPS	0.071	-0.002	0.117	0.056	0.76	
SHG	0.683	0.237	0.201	0.384	0.052	0.787

Table S5b. The Fornell-Larcker criterion in discriminant validity assessment (Thailand)

	MHB	CHG	HGI	PHB	PPS	SHG
MHB	0.869					
CHG	0.006	0.876				
HGI	0.231	-0.388	0.831			
PHB	0.712	0.003	0.175	0.905		
PPS	-0.078	-0.005	0.116	-0.061	0.793	
SHG	-0.367	-0.388	0.226	-0.331	0.074	0.839

Table S5c. The Fornell-Larcker criterion in discriminant validity assessment (Vietnam)

	MHB	CHG	HGI	PHB	PPS	SHG
MHB	0.847					
CHG	0.369	0.894				
HGI	0.268	-0.091	0.818			
PHB	0.103	0.087	0.165	0.839		
PPS	0.366	0.043	0.249	-0.001	0.815	
SHG	0.556	0.577	0.155	0.087	0.157	0.886
